# Probing interlayer shear thermal deformation in atomically-thin van der Waals layered materials

**DOI:** 10.1038/s41467-022-31682-w

**Published:** 2022-07-09

**Authors:** Le Zhang, Han Wang, Xinrong Zong, Yongheng Zhou, Taihong Wang, Lin Wang, Xiaolong Chen

**Affiliations:** 1 Department of Electrical and Electronic Engineering, Southern University of Science and Technology, 1088 Xueyuan Avenue, 518055 Shenzhen, P.R. China; 2 Key Laboratory of Flexible Electronics (KLOFE) & Institute of Advanced Materials (IAM), Nanjing Tech University (Nanjing Tech), 30 South Puzhu Road, 211816 Nanjing, P.R. China

**Keywords:** Mechanical engineering, Two-dimensional materials

## Abstract

Atomically-thin van der Waals layered materials, with both high in-plane stiffness and bending flexibility, offer a unique platform for thermomechanical engineering. However, the lack of effective characterization techniques hinders the development of this research topic. Here, we develop a direct experimental method and effective theoretical model to study the mechanical, thermal, and interlayer properties of van der Waals materials. This is accomplished by using a carefully designed WSe_2_-based heterostructure, where monolayer WSe_2_ serves as an in-situ strain meter. Combining experimental results and theoretical modelling, we are able to resolve the shear deformation and interlayer shear thermal deformation of each individual layer quantitatively in van der Waals materials. Our approach also provides important interlayer coupling information as well as key thermal parameters. The model can be applied to van der Waals materials with different layer numbers and various boundary conditions for both thermally-induced and mechanically-induced deformations.

## Abstract (web summary)

Van der Waals materials exhibit unique thermomechanical properties, but interlayer deformations are usually challenging to measure. Here, the authors exploit the strain-dependent optical properties of monolayer WSe_2_ to quantitatively probe the interlayer shear thermal deformations and interlayer coupling in phosphorene and hexagonal boron nitride.

## Introduction

Triggered by the growing need of developing next-generation semiconductor devices, mechanical engineering has been moved forward from traditional semiconductors to van der Waals (vdW) materials due to their unique layered structures^1,2^. Through lattice deforming, the electronic structure of vdW materials can be tuned significantly, giving rise to intriguing physical phenomena and applications, such as shear-strain-generated pseudo magnetic fields^3^, one-dimensional moiré potentials^4^, confined states in soliton networks^5^, and actively variable-spectrum optoelectronics^6^. Mechanical approaches have been widely used to introduce compressive and tensile strain (lattice deformation) in vdW materials, including substrate engineering with nanopillars^7–9^, generating nanobubbles in vdW materials^10–13^, bending flexible substrates^14–16^, and utilizing the thermal expansion coefficient (TEC) mismatch between vdW materials and substrates^17–19^. Although much progress has been achieved in the mechanical engineering of vdW materials, investigations on their thermomechanical properties are scarce. Besides, understanding of the micro-mechanism of interlayer deformation when reacting to temperature variation lies at the heart of thermal engineering of vdW materials.

**Fig. 1:**
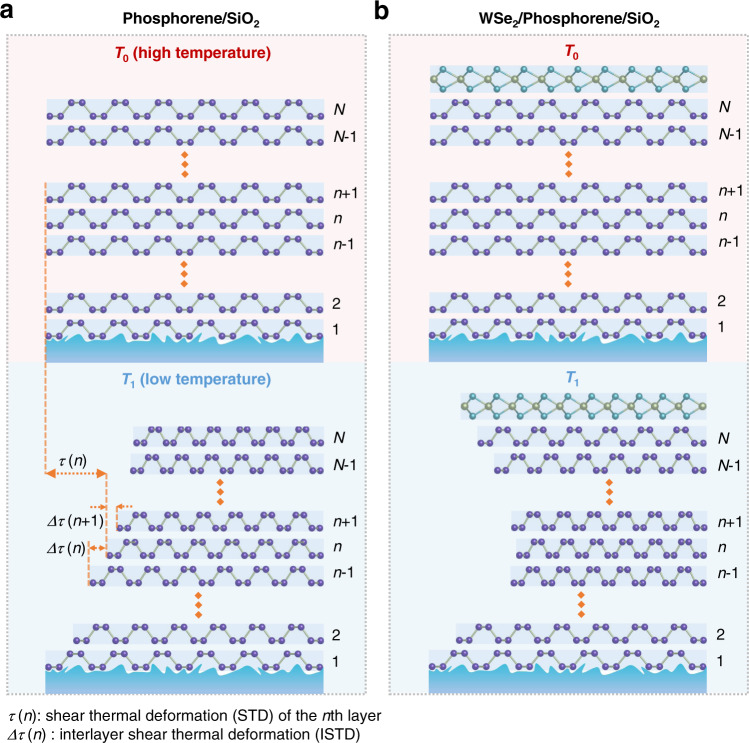
Shear thermal deformation (STD) and interlayer shear thermal deformation (ISTD) in van der Waals materials and heterostructures. **a** ISTD model of an *N*-layer phosphorene/SiO_2_ system when temperature decreases from *T*_0_ to *T*_1_. **b** ISTD model of a WSe_2_/*N*-layer phosphorene/SiO_2_ system when temperature decreases from *T*_0_ to *T*_1_. Here, *N* is the total layer number of phosphorene. *n* is the *n*-th (1 ≤ *n* ≤ *N*) phosphorene layer counting from bottom*. τ* (*n*) is the STD of the *n-*th phosphorene and *Δτ* (*n*) is the ISTD between the *n*-th and *(n−*1)-th phosphorene from *T*_0_ to *T*_1_.

Since vdW materials are always supported by substrates, their thermomechanical properties are considered based on a whole vdW-materials/substrate system. vdW materials and substrates generally possess distinct TEC, leading to distinct intrinsic thermal deformation when temperature changes. Figure 1a shows the schematic diagram of thermal deformation in an *N*-layer vdW-material/SiO_2_ system from *T*_0_ to *T*_1_. Considering the strong clamping effect between SiO_2_ and vdW materials^18^, the deformation of the bottom layer (*n* = 1) is nearly equal to that of SiO_2_. Yet the top layer (*n* = *N*) is almost free from the clamping effect and exhibits intrinsic thermal deformation of vdW materials when *N* is large enough. In this case, the relaxation from layer to layer through interlayer interaction results in the in-plane lattice deformation difference between adjacent layers. For clarity, we define the shear thermal deformation (STD) *τ* and interlayer shear thermal deformation (ISTD) Δ*τ* in Fig. 1a. Here, *τ* is the in-plane lattice deformation induced by temperature variation from *T*_0_ (high temperature) to *T*_1_ (low temperature) and Δ*τ* is the in-plane lattice deformation difference between adjacent layers. However, owing to the lack of proper characterization technique, precise measurements of STD and ISTD layer by layer in vdW materials have not been reported yet.

In this work, we choose phosphorene and hexagonal boron nitride (hBN) as the representative experimental subjects due to their exceptional thermal and mechanical properties^18,20–22^. Through monitoring the temperature-dependent photoluminescence (PL) spectra of delicately designed WSe_2_-based vdW heterostructures, where monolayer WSe_2_ serves as an in-situ “strain meter”, the mechanical behaviors of vdW materials are reflected conveniently. Taking account of interlayer interactions at both homo- and hetero-interfaces and the Young’s modulus and TEC of phosphorene and WSe_2_, we establish an effective ISTD model which allows us to access the layer-dependent STD and ISTD in phosphorene. The schematic diagram in Fig. 1b illustrates the thermal deformation reaction of phosphorene and WSe_2_ layers in the WSe_2_/phosphorene/SiO_2_ system from *T*_0_ to *T*_1_. Through fitting the experimental results, we can extract the interlayer coupling coefficients at phosphorene/phosphorene homo-interface and WSe_2_/phosphorene hetero-interface. Besides, key thermal parameters of vdW materials, such as TEC, are extracted from the model.

## Results

### Design of vdW heterostructures for ISTD studies

**Fig. 2:**
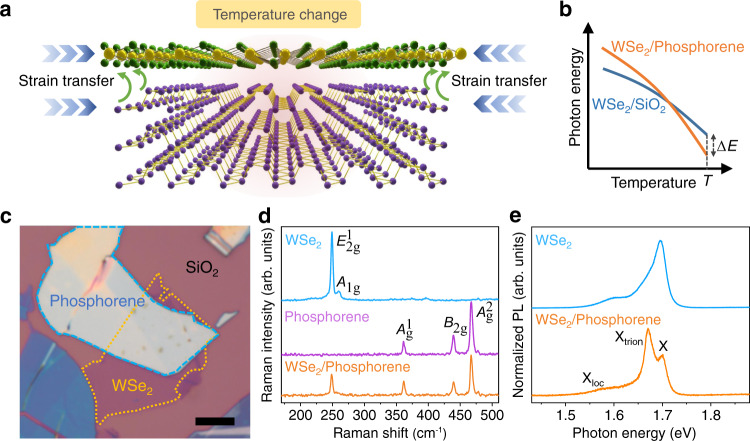
WSe_2_/phosphorene heterostructure for STD and ISTD investigations. **a** Monolayer WSe_2_ serves as a strain meter to probe the thermal deformation of the top phosphorene layer. Here, the blue arrows indicate that the in-plane lattice contraction when temperature decreases. The green arrows describe the strain transfer from phosphorene to WSe_2_ through interlayer interactions. **b** Schematic evolution trends of photon energy in WSe_2_/phosphorene/SiO_2_ and WSe_2_/SiO_2_ systems as a function of temperature. Photon energy difference (Δ*E*) between WSe_2_/phosphorene/SiO_2_ and WSe_2_/SiO_2_ at temperature *T* can directly reflect the thermal deformation of WSe_2_. **c** Optical image of a WSe_2_/phosphorene heterostructure. The scale bar is 5 μm. **d** Raman spectra of isolated monolayer WSe_2_, isolated phosphorene, and WSe_2_/phosphorene heterostructure at room temperature. **e** Photoluminescence (PL) spectra of isolated monolayer WSe_2_ and WSe_2_/phosphorene heterostructure at 180 K. Here, X_loc_, X_trion_ and X denotes the localized, charged and neutral exciton of monolayer WSe_2_, respectively.

We choose monolayer WSe_2_ and phosphorene as building blocks of vdW heterostructures for STD and ISTD investigations for three reasons. First, phosphorene has been predicted to exhibit a large TEC of between 6.3 × 10^−6^ and 53 × 10^−6^ K^−1^ at room temperature^23–25^, which stands out from the family of vdW materials and is at least one order of magnitude larger than that of SiO_2_ (~0.5 × 10^−6^ K^−1^)^26,27^. Such a large TEC is expected to cause significant thermal deformation and corresponding effect on the physical properties of phosphorene and its adjacent 2D materials. Second, monolayer WSe_2_ is a flexible direct-bandgap semiconductor with very high luminescence efficiency^28^. Its strain-sensitive optical and electronic properties have been widely investigated^16,17,28–30^. Thus, we can use monolayer WSe_2_ as a convenient sensing layer to monitor the thermal deformation of phosphorene as illustrated in Fig. 2a. Third, phosphorene and WSe_2_ show strong coupling at their interface, which could enable efficient strain transfer from phosphorene to WSe_2_ through interlayer interactions (Fig. 2a)^31^.

To quantitatively investigate the STD and ISTD of phosphorene, we monitor the temperature-dependent PL photon energy of WSe_2_/phosphorene/SiO_2_ and use that of WSe_2_/SiO_2_ as a reference system, since the TEC of SiO_2_ can be neglected compared with that of phosphorene^23–26^. Even though WSe_2_ in WSe_2_/SiO_2_ and WSe_2_/phosphorene/SiO_2_ experiences different dielectric environments which could affect the exciton binding energies of WSe_2_, in this study we are focusing on Δ*E’* = Δ*E*(*T*_1_) − Δ*E*(*T*_0_), where Δ*E*(*T*) is the relative shift of photon energy in WSe_2_/phosphorene/SiO_2_ compared with that in WSe_2_/SiO_2_ at temperature *T* (see Fig. 2b). The effect of dielectric environment plays a minor role in determining Δ*E’* as shown in Supplementary Fig. 1 and Supplementary Note 1. As illustrated in Fig. 2b, Δ*E’* directly reflects the STD of WSe_2_ in the temperature range from *T*_0_ to *T*_1_. Considering the interlayer coupling effect at the WSe_2_/phosphorene interface, information of STD and ISTD in phosphorene layers can be extracted.

### Characterizations of WSe_2_/phosphorene heterostructures

Figure 2c shows the optical image of a WSe_2_/phosphorene heterostructure, assembled by the PDMS-assisted dry-transfer method^32^. To achieve a high-quality vdW interface, all exfoliation and transfer processes were performed in a N_2_-filled glove box. The cross-sectional scanning transmission electron microscopy (STEM) image and elemental mapping demonstrate a clean and amorphous-phase-free WSe_2_/phosphorene interface (see Supplementary Fig. 2 and Supplementary Note 2). The Raman spectra collected from isolated monolayer WSe_2_, isolated phosphorene, and WSe_2_/phosphorene heterostructure are displayed in Fig. 2d, respectively. The characteristic phonon vibration modes of the monolayer WSe_2_ (${E}_{2{{{{{\rm{g}}}}}}}^{1}$)^33^ and few-layer phosphorene (${A}_{{{{{{\rm{g}}}}}}}^{1}$, *B*_2g_, and ${A}_{{{{{{\rm{g}}}}}}}^{2}$)^34–36^ are all observed in the heterostructure region. Figure 2e shows the PL spectra of the isolated WSe_2_ and WSe_2_/phosphorene heterostructure at 180 K, where layer numbers of the phosphorene are 50 (~27.5 nm) determined by atomic force microscope. Three pronounced photon emission peaks, located at 1.59, 1.67, and 1.70 eV, are observed. They can be attributed to localized exciton (X_loc_), charged exciton (X_trion_), and neutral exciton (X)^37,38^, respectively. The X_trion_ emission is more pronounced in WSe_2_/phosphorene heterostructure which can be attributed to the charge transfer at vdW interface^39^.

**Fig. 3:**
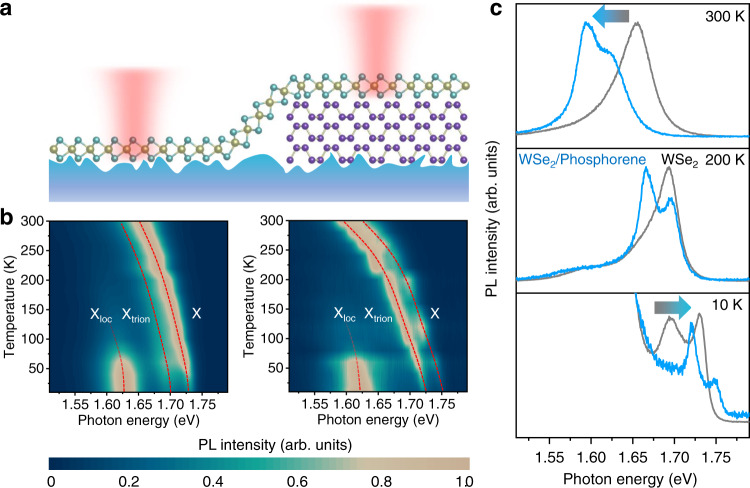
Temperature-dependent PL characterizations. **a** Schematic diagram of PL characterizations on WSe_2_/SiO_2_ and WSe_2_/phosphorene/SiO_2_ regions. **b** PL of neutral exciton (X), charged exciton (X_trion_) and localized exciton (X_loc_) in isolated WSe_2_ on SiO_2_ substrate and WSe_2_/phosphorene heterostructure from 10 to 300 K. The red dashed lines serve as guide lines. **c** The normalized PL spectra of isolated WSe_2_ (gray lines) and WSe_2_/phosphorene heterostructure (blue lines) at 10, 200, and 300 K. Here, the gray-blue arrows indicate that the photon energy of X and X_trion_ in WSe_2_/phosphorene/SiO_2_ heterostructure is blue-shifted/red-shifted relative to those in WSe_2_/SiO_2_.

Then we explore the temperature-dependent properties of PL in the isolated WSe_2_ on SiO_2_ and WSe_2_/phosphorene heterostructure (see the schematic diagram in Fig. 3a). As shown in Fig. 3b, both X and X_trion_ show energy shift when temperature changes. Moreover, X and X_trion_ of WSe_2_/phosphorene heterostructure go through a greater shift than those of the isolated WSe_2_ on SiO_2_, indicating that additional strain is induced to the WSe_2_ on phosphorene when temperature changes. Figure 3c further compares the normalized PL of isolated WSe_2_ and WSe_2_/phosphorene heterostructure at three representative temperatures. The photon energy of X and X_trion_ in WSe_2_/phosphorene heterostructure is larger than that in WSe_2_ at 10 K, the same at 200 K, and smaller at 300 K. With temperature changing, the top phosphorene layer performs greater in-plane lattice deformation than SiO_2_. Therefore, an additional strain is transferred into WSe_2_ on phosphorene through vdW interlayer interactions, leading to the greater shift of photon energy relative to that of WSe_2_ on SiO_2_. According to previous research results, tensile/compressive strain in monolayer WSe_2_ will reduce/enlarge its optical bandgap^16,17,28–30^, showing excellent agreement with our observations.

### ISTD model and experimental measurement

To confirm our expectations, an ISTD model considering interlayer coupling effect is established to quantitatively determine the STD and ISTD in vdW materials. When temperature changes, the in-plane interlayer interaction is generated between adjacent layers due to lattice deformation mismatch (i.e., the ISTD). This produces additional in-plane stress in individual layer. Here, the in-plane stress is linked to ISTD through a proportionality factor, which is defined as the interlayer coupling coefficient (*c*) between adjacent layers. *c*_p_ and *c*_h_ denote the interlayer coupling coefficient at phosphorene/phosphorene homo-interface and WSe_2_/phosphorene hetero-interface, respectively.

Taking *T*_0_ as the initial state, the strain in each layer is 0 (see Fig. 1). As shown in Fig. 1a, the STD of the *n*-th phosphorene (1 ≤ *n* ≤ *N*, counting from the bottom layer) from *T*_0_ to *T*_1_ is noted as *τ*(*n*). When 2 ≤ *n* ≤ *N*−1, the *n*-th phosphorene interacts with the (*n*−1)-th as well as with the (*n* + 1)-th phosphorene layers. Therefore, *τ*(*n*) satisfies the following equation at *T*_1_ [Eq. 1]:

1 $${c}_{{{{{{\rm{p}}}}}}}(\varDelta \tau (n+1)-\varDelta \tau (n))={\gamma }_{{{{{{\rm{p}}}}}}}(\tau (n)-{\tau }_{{{{{{\rm{p}}}}}}})\;\;\;\;\;2\le n\le N-1$$

Here, ∆*τ*(*n*) is the ISTD between the *n*-th and (*n−*1)-th phosphorene (Fig. 1a). *γ*_p_ is the Young’s modulus of phosphorene, which is around 60 GPa according to previous works^40^. *τ*_p_ is the thermally-induced intrinsic deformation of phosphorene, which is a constant depending on the TEC of phosphorene.

The cases of phosphorene/SiO_2_ (Fig. 1a) and WSe_2_/phosphorene/SiO_2_ (Fig. 1b) share the same boundary condition for *n* = 1 (at phosphorene/SiO_2_ interface). When *n* = 1, considering the strong clamping effect between the bottom phosphorene and SiO_2_ substrate^18^ as well as the tiny TEC of SiO_2_^26,27^, an approximation can be made that the STD of the bottom phosphorene is negligible, that is, *τ*(1) = 0. When *n* = *N*, the mechanical behaviors of the top phosphorene are totally different in phosphorene/SiO_2_ and WSe_2_/phosphorene/SiO_2_ systems. In WSe_2_/phosphorene/SiO_2_ system, the interlayer interaction at phosphorene/WSe_2_ interface and the mechanical and thermal properties of WSe_2_ have direct impact on the STD of phosphorene layers. Please refer to Supplementary Note 3 for the details, where the interlayer coupling coefficient between phosphorene and WSe_2_ (*c*_h_), the Young’s modulus of WSe_2_ $({\gamma }_{{{{{{\rm{WS}}}}}}{{{{{{\rm{e}}}}}}}_{2}})$ and thermally-induced intrinsic deformation of WSe_2_ $({\tau }_{{{{{{\rm{WS}}}}}}{{{{{{\rm{e}}}}}}}_{2}})$ are introduced. Here, *c*_h_ is calculated as 2.72 × 10^11 ^Pa, ${\gamma }_{{{{{{\rm{WS}}}}}}{{{{{{\rm{e}}}}}}}_{2}}$ is assigned to be 120 GPa according to previous reports^41–45^, and ${\tau }_{{{{{{\rm{WS}}}}}}{{{{{{\rm{e}}}}}}}_{2}}$ is extracted to be −0.17% at 10 K (see Supplementary Note 4).

**Fig. 4:**
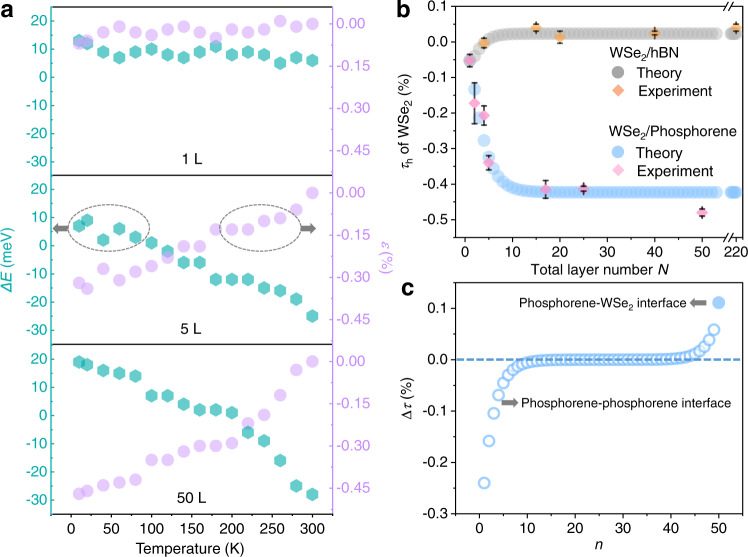
Extracting STD and ISTD from experiment results and theoretical modelling. **a** Cyan symbols show the temperature-dependent photon energy difference (Δ*E*) between WSe_2_/phosphorene and isolated WSe_2_ at phosphorene layer numbers of 1, 5, and 50, respectively. Violet symbols shows the experimentally measured strain of WSe_2_ as a function of temperature. **b** From 300 to 10 K, experimentally measured and theoretically fitted STD of WSe_2_ (*τ*_h_) in WSe_2_/phosphorene and WSe_2_/hBN systems with different total layer number *N*. Here, for each sample, PL signals are collected from at least three spots near the center of heterostructures. The average value (pink/orange symbols) and standard deviation (error bar, black solid line) of *τ*_h_ (*Ν*) are therefore obtained from the measured ∆*E*. **c** Theoretically calculated layer-dependent ISTD (Δ*τ*) in the WSe_2_/50-layer phosphorene/SiO_2_ system. Here, the empty circles denote Δ*τ* at phosphorene-phosphorene interface while the filled circle denotes Δ*τ* at phosphorene-WSe_2_ interface.

To confirm the validity of our theory, we experimentally characterize WSe_2_/phosphorene heterostructures with various phosphorene layer number *N* from 1 to 50. Figure 4a shows measured ∆*E* as a function of temperature at three representative layer numbers of 1, 5, and 50 (cyan symbols), respectively. Then, taking advantage of *η* = −100 meV/% (see Supplementary Fig. 3, Supplementary Table 1 and Supplementary Note 5), where *η* is the coefficient of strain-induced energy shift in monolayer WSe_2_, the additional in-plane strain of WSe_2_ in the heterostructure *ε* = ∆*E*/*η* can be extracted (violet symbols in Fig. 4a). The fitting results at other *η* values are further shown and compared in Supplementary Table 2.

Taking 300 K as the initial temperature *T*_0_ and 10 K as the final temperature *T*_1_, the STD of WSe_2_ on *N*-layer phosphorene, *τ*_h_(*N*) = (∆*E*(*T*_1_) − ∆*E*(*T*_0_))/*η*, is shown in Fig. 4b. The measured STD (pink symbols) agrees well with the theoretical fitting results (blue symbols), with fitting parameters *c*_p_ = 3.41 × 10^11^ Pa and *τ*_p_ = −0.71%. The error analysis of the theoretical fitting results is provided in Supplementary Fig. 4 and Supplementary Note 6. Here, the larger interlayer coupling coefficient *c*_p_ at phosphorene/phosphorene homo-interfaces than *c*_h_ at WSe_2_/phosphorene hetero-interfaces indicates the stronger coupling at phosphorene/phosphorene homo-interfaces. When *N* > 15, *τ*_h_ is almost independent of layer number and reaches the minimum value of −0.42%. This phenomenon is in accordance with our expectations since the substrate clamping effect is weaker for top phosphorene layers when *N* is larger. Utilizing the fitting results above, we are able to calculate the *n*-dependent STD and ISTD quantitatively in WSe_2_/phosphorene/SiO_2_ systems with various total layer number *N*. Figure 4c shows the *n*-dependent ISTD (*∆τ*) in a WSe_2_/50-layer phosphorene heterostructure. With *n* increasing, there appears a crossover point where the negative *∆τ* turns into positive. The slope is steeper near the phosphorene/SiO_2_ and WSe_2_/phosphorene hetero-interfaces due to strong mismatch of in-plane strain. Besides, we can access the layer-dependent in-plane force in phosphorene and WSe_2_ layers as shown in Supplementary Fig. 5. The in-plane force is large near phosphorene/SiO_2_ and WSe_2_/phosphorene interfaces (at order of 0.1 N m^−1^) while vanishes in the central region.

**Fig. 5:**
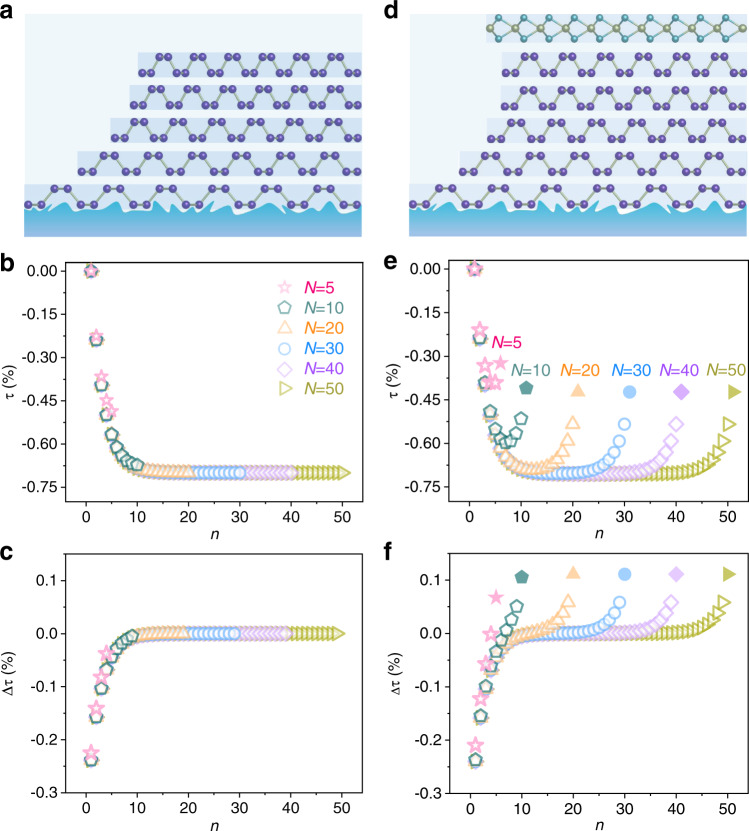
Resolving STD and ISTD of individual layer in phosphorene/SiO_2_ and WSe_2_/phosphorene/SiO_2_ systems. **a** Schematic diagram of 5-layer phosphorene/SiO_2_ under thermomechanical deformation at low temperature. **b**, **c** Theoretically calculated layer-dependent *τ* (**b**) and ∆*τ* (**c**) in phosphorene/SiO_2_ at representative layer numbers *N* = 5, 10, 20, 30, 40 and 50. **d** Schematic diagram of WSe_2_/5-layer phosphorene/SiO_2_ at at low temperature. **e**, **f** Theoretically calculated layer-dependent *τ* (**e**) and ∆*τ* (**f**) in WSe_2_/phosphorene/SiO_2_ at representative layer numbers *N* = 5, 10, 20, 30, 40, and 50. Here, the empty symbols denote values in phosphorene while the filled symbols denote values in WSe_2_.

Utilizing the fitting results of *c*_p_ = 3.41 × 10^11 ^Pa and *τ*_p_ = −0.71%, STD and ISTD of phosphorene in phosphorene/SiO_2_ system can be also calculated (see Supplementary Note 3). Here, we compare the calculation results of phosphorene/SiO_2_ and WSe_2_/phosphorene/SiO_2_ systems with different *N* in Fig. 5. Figure 5a and d are the schematic diagrams of the two cases at *N* = 5. In phosphorene/SiO_2_ system, STD of phosphorene (*τ*) decreases monotonously and nonlinearly with *n* (see Fig. 5b). When *N* > *n* > 15, *τ* is almost independent of *n* and reaches the minimum value of −0.71%, which approaches the intrinsic thermal deformation of phosphorene at 10 K. On the other hand, in WSe_2_/phosphorene/SiO_2_ system, STD of phosphorene initially decreases and then increases from bottom to top, revealing the non-uniform deformation near the WSe_2_/phosphorene interface (Fig. 5e). When *N* is large enough, *τ* reaches the minimum value −0.71% in the middle region (17 < *n* < 28 for *N* = 50) and increases to −0.53% at *n* = *N*. In addition, *∆τ* of phosphorene increases monotonously with *n* and approaches zero when *N* > *n* > 15 in phosphorene/SiO_2_ system (Fig. 5c), while in WSe_2_/phosphorene/SiO_2_ system, *∆τ* turns into positive values near the WSe_2_/phosphorene interface due to the small TEC and large Young’s modulus of WSe_2_ (Fig. 5f). The distinctions between the two cases directly reflect the interlayer coupling effect between WSe_2_ and phosphorene.

### TEC of phosphorene

To our best knowledge, experimental studies on the TEC (*α*) of atomically-thin vdW layered materials are scarce, all of which are based on the high-temperature X-ray diffraction technique^23,25,46^, temperature-dependent Raman and electron energy-loss spectroscopy^21,27^. In the following section, we are going to show that the additional strain measured in WSe_2_/phosphorene heterostructures can be utilized to achieve this goal.

**Fig. 6:**
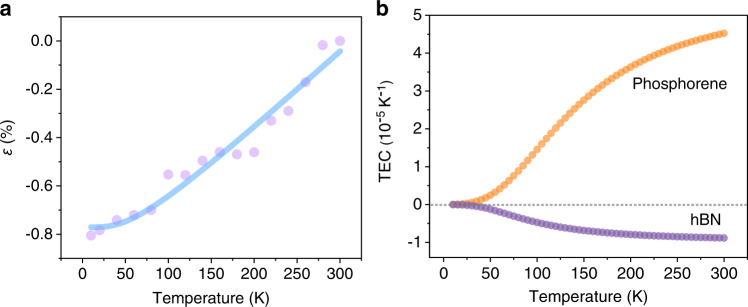
Thermal parameters of phosphorene and hBN. **a** Experimentally measured (violet symbols) and theoretically fitted (blue solid line) intrinsic thermal strain of phosphorene as a function of temperature. **b** Extracted temperature-dependent thermal expansion coefficient (TEC) of phosphorene and hBN.

Utilizing the temperature-dependent additional strain of WSe_2_ in the WSe_2_/50-layer phosphorene (Fig. 4a), the intrinsic thermal strain of phosphorene as a function of temperature can be extracted through the ISTD model (violet symbols in Fig. 6a). A local band average approach under Debye approximation has declared that the temperature-dependent TEC is proportional to the specific heat (*C*_v_) and can be expressed as^47^ [Eq. 2]:

2 $$\alpha (T)=A{(T/{\theta }_{{{{{{\rm{D}}}}}}})}^{3}{\int }_{0}^{{\theta }_{{{{{{\rm{D}}}}}}}/T}\frac{{x}^{4}{e}^{x}}{{({e}^{x}-1)}^{2}}dx$$

where *A* is a constant and *θ*_*D*_ is the Debye temperature. Then the thermal strain of the phosphorene can be obtained by integrating TEC [Eq. 3]:

3 $$\varepsilon (T)=\int \alpha (T)dT$$

According to previous reports, *θ*_D_ = 600 K is used to conduct the fitting^48^. As shown in Fig. 6a, the fitted results (blue solid line) match well with the experimental results (violet symbols) with parameter *A* = 1.64 × 10^−4 ^K^−1^, indicating the validity of our ISTD model. Figure 6b shows the extracted TEC of phosphorene from our theoretical model. The TEC is very small at low temperature and increases to 4.52 × 10^−5 ^K^−1^ at 300 K. The obtained TEC ~ 4.52 × 10^−5 ^K^−1^ at room temperature agrees quite well with previously reported values at high temperature (>300 K)^23–25^.

### STD and TEC of hBN

Distinct from phosphorene, hBN possesses a negative TEC instead, whose absolute value is an order of magnitude smaller than that of phosphorene according to previous studies^21,46^. Therefore, to confirm the validity of our ISTD model, we repeat experiments based on WSe_2_/hBN heterostructures. The layer-dependent STD of hBN *τ* (*n*) is extracted and plotted in Fig. 4b, with fitting parameters *c*_hBN_ = 5.03 × 10^11^ Pa and *τ*_hBN_ = 0.17%. STD of WSe_2_ in WSe_2_/hBN and WSe_2_/phosphorene shows distinct trend with *N*. This phenomenon is in accordance with the small and negative TEC of hBN. Then, the WSe_2_/220-layer hBN heterostructure is adopted to extract TEC of hBN utilizing *θ*_D_ = 410 K^46^. The temperature-dependent TEC of hBN is plotted in Fig. 6b (purple symbols). The extracted TEC is −8.83 × 10^−6 ^K^−1^ at room temperature, showing good agreement with reported values^21,46^. Therefore, the investigation of hBN further confirms the validity of our ISTD model and the effectiveness of the technique for sensing thermal properties of vdW layered materials.

## Discussion

At last, we discuss the novelty of this work and the distinction from previous works^13,49,50^. First, we provide a smart strategy, the WSe_2_-based heterostructure, to investigate the mechanical, thermal, and interlayer coupling properties of vdW materials. Second, the ISTD model can quantitatively resolve the interlayer deformation in individual layers of vdW materials and heterostructures with various layer numbers. Third, the model can provide important interlayer coupling information, such as the interlayer coupling coefficients and in-plane force at phosphorene/phosphorene homo-interface and WSe_2_/phosphorene hetero-interface. Fourth, the intrinsic-thermal-deformation-induced strain is more stable, reversible, and controllable compared with mechanical bending/stretching, allowing us to provide a clearer physical picture of interlayer shear deformation and extract key thermal parameters accurately. Last, the model can be applied and extended to various deformation situations (both thermally-induced and mechanically-induced deformations) with various boundary conditions, which can be easily modified and used by other researchers. Hence, we believe the smart experimental methodology, the ISTD model, the clear physical picture and the interlayer coupling information provided in this work will inspire thermomechanical engineering in vdW materials and be beneficial to the scientific community of 2D materials.

## Methods

### Sample preparation

Monolayer and few-layer phosphorene/hBN flakes were mechanically exfoliated from bulk crystals (purchased from HQ graphene) onto 285 nm SiO_2_/Si substrate through the standard scotch tape method. To avoid material degradation, the exfoliation process was carried out in a N_2_-filled glovebox (MIKROUNA-Universal Series) with O_2_ and H_2_O concentrations smaller than 0.01 ppm. The monolayer WSe_2_ was exfoliated onto PDMS substrate. Then the WSe_2_/phosphorene and WSe_2_/hBN heterostructures were assembled using the PDMS-assisted dry-transfer method in the glovebox. We intentionally left half WSe_2_ flake on SiO_2_ as a reference to extract the thermal deformation of phosphorene and hBN. To improve the interlayer coupling between WSe_2_ and phosphorene/hBN, the samples are annealed at 200 °C for 10 min inside the glovebox.

### Optical characterizations

To carry out the temperature-dependent PL measurements, the samples were loaded in a He-flow closed-cycle cryostat (Advanced Research System) with a high vacuum of ~2 × 10^−6 ^Torr. A 532 nm laser was used as the excitation source and focused on the sample by a 50× objective lens (NA = 0.5). The laser power was kept at a low value of 25 μW to avoid laser-induced damage and heating effect. The PL signals were dispersed by an Andor SR-500i-D2 spectrometer with a 150 g/mm grating and detected using an Andor iVac 316 CCD. As for the Raman measurements, which were conducted at room temperature, a 600 g/mm grating was used.

## Supplementary information

Supplementary information

Peer Review File

## Data Availability

Relevant data supporting the key findings of this study are available within the paper and the Supplementary Information file. All raw data generated during the current study are available from the corresponding authors upon request.
