# Supplementary material for: Probing interlayer shear thermal deformation in atomically-thin van der Waals layered materials
Source: Nat Commun. 2022 Jul 9;13:3996. doi: 10.1038/s41467-022-31682-w (PMC9271035; doi:10.1038/s41467-022-31682-w)
Supplement: Supplementary file 1 — Supplementary information [file 41467_2022_31682_MOESM1_ESM.pdf]

Probing interlayer shear thermal deformation in atomically-thin van der Waals layered materials

AUTHORS

Le Zhang[1], Han Wang[1], Xinrong Zong[2], Yongheng Zhou[1], Taihong Wang[1], Lin Wang[2]*, Xiaolong Chen[1]*

[1] Department of Electrical and Electronic Engineering, Southern University of Science and Technology, 1088 Xueyuan Avenue, Shenzhen 518055, P.R. China, [2] Key Laboratory of Flexible Electronics (KLOFE) & Institute of Advanced Materials (IAM), Nanjing Tech University (Nanjing Tech), 30 South Puzhu Road, Nanjing 211816, P.R. China. *Email: iamlwang@njtech.edu.cn; chenxl@sustech.edu.cn

These authors contributed equally: Le Zhang, Han Wang

These authors jointly supervised this work: Xiaolong Chen, Lin Wang

Supplementary Note 1

Monolayer WSe_2 in WSe_2/SiO_2 and WSe_2/phosphorene/SiO_2 systems experiences different dielectric environments, since the static dielectric constant of SiO_2 (~3.9)[1] is smaller than that of BP (~6)[2]. It is well known that the exciton binding energy of low-dimensional materials is sensitive to the dielectric environment. However, in this work, it is the $\Delta E' = \Delta E_{10K} - \Delta E_{300K} = (E_{WSe_2/BP}(10K) - E_{WSe_2}(110K)) - (E_{WSe_2/BP}(300K) -$

E_{WSe_2}(300K)) that is used to determine the thermal deformation of WSe_2 and phosphorene $\tau \propto \Delta E'$. Here, $E_{WSe_2/BP}$(300K) and E_{WSe_2}(300K) are PL photon energies of WSe_2/BP/SiO_2 and WSe_2/SiO_2 at 300 K, respectively. $E_{WSe_2/BP}$(10K) and E_{WSe_2}(10K) are PL photon energies of WSe_2/BP/SiO_2 and WSe_2/SiO_2 at 10 K, respectively. As we can see from the above equation, the effect of exciton binding energy (which strongly depends on the dielectric environment) is probably canceled when calculating ΔE. Besides, we further experimentally demonstrate that the dielectric environment has negligible impact on ΔE.

In this experiment, we fabricated two BP/WSe_2/SiO_2 (WSe_2 is sandwiched by BP and SiO_2) heterostructure samples (see Supplementary Fig. 1a and 1b), where the monolayer WSe_2 in WSe_2/SiO_2 and BP/WSe_2/SiO_2 experiences different dielectric environments (see Supplementary Fig. 1b). In addition, the in-plane lattice deformation of WSe_2 in both WSe_2/SiO_2 and BP/WSe_2/SiO_2 heterostructures can be regarded as zero due to the strong clamping effect of SiO_2 substrates. We monitored the temperature-dependent photon energy of BP/WSe_2/SiO_2 using that of WSe_2/SiO_2 as a reference. Here, ΔE is the relative shift of photon energy in BP/WSe_2/SiO_2 (or WSe_2/BP/SiO_2) compared with that in WSe_2/SiO_2, while $\Delta E'$ is the difference of ΔE between 300 and 10 K ($\Delta E' = \Delta E_{10K} - \Delta E_{300K}$). The temperature-dependent ΔE of the two BP/WSe_2/SiO_2 samples (the orange and olive scatters) and a WSe_2/BP/SiO_2 sample (the violet scatters) are displayed in Supplementary Fig. 1c. $\Delta E'$ in BP/WSe_2/SiO_2 heterostructures are extracted to be 0 and -3 meV, which are negligible compared to the large value ~ 47

meV in $WSe_2/BP/SiO_2$ heterostructures. This experiment proves that different dielectric environments in WSe_2/SiO_2 and $BP/WSe_2/SiO_2$ heterostructures do not affect the determination of $\Delta E'$ and τ.

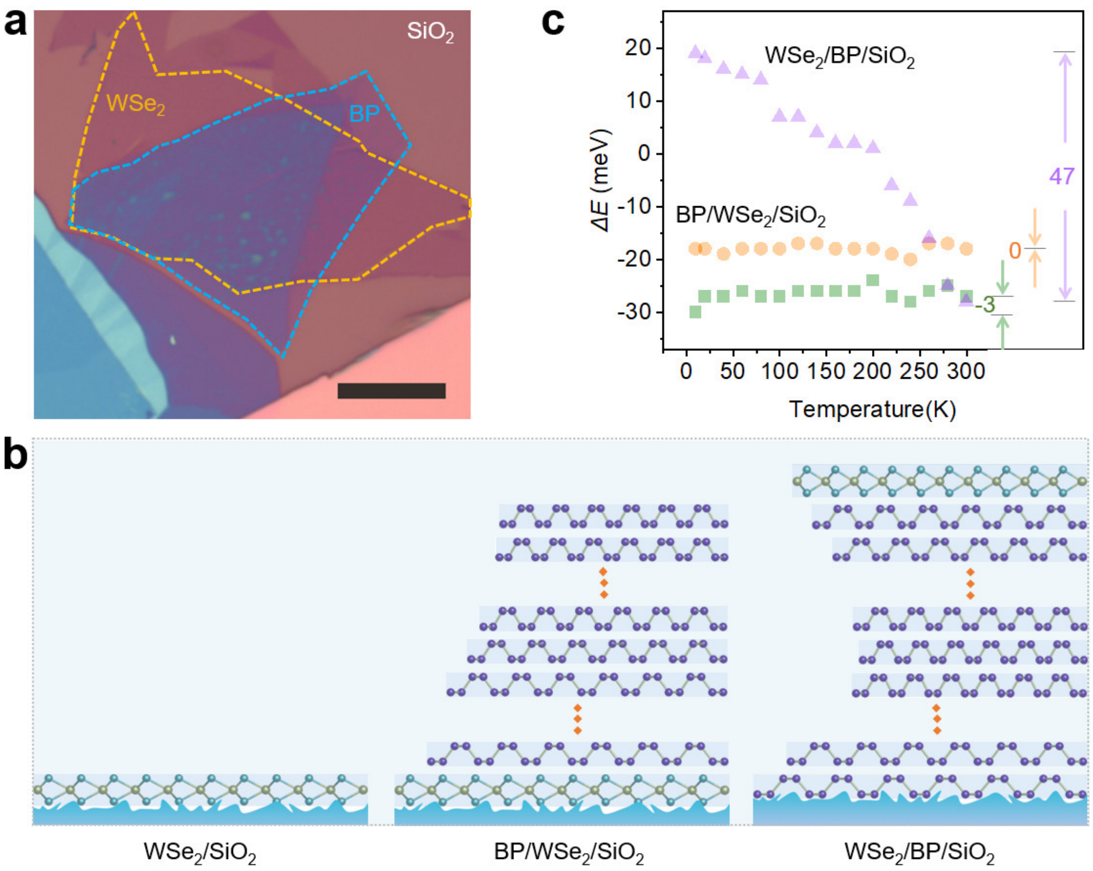

Supplementary Figure 1. Effect of dielectric environment on photon energy difference. a Optical image of a $BP/WSe_2/SiO_2$ heterostructure. The scale bar is 10 μm. **b** Schematic diagrams of WSe_2/SiO_2, $BP/WSe_2/SiO_2$, and $BP/WSe_2/SiO_2$ at low temperature. **c** Temperature-dependent photon energy difference (ΔE) between WSe_2 in heterostructures and isolated WSe_2 on SiO_2. Here, the orange and olive scatters denote $BP/WSe_2/SiO_2$ systems and the violet scatters denote $WSe_2/BP/SiO_2$ system.

Supplementary Note 2

We have fabricated a graphene/WSe_2/ phosphorene heterostructure for cross-sectional scanning transmission electron microscopy (STEM) characterizations. Here, graphene serves as the protective layer to avoid damage of WSe_2/ phosphorene structures when preparing the STEM cross-sectional sample. The cross-sectional STEM image shows a clean interface of WSe_2/phosphorene. Supplementary Fig. 2b shows the elemental mapping for carbon (C), phosphorus (P), selenium (Se), and tungsten (W). The STEM image and elemental mapping demonstrate a clean and amorphous-phase-free WSe_2/phosphorene interface.

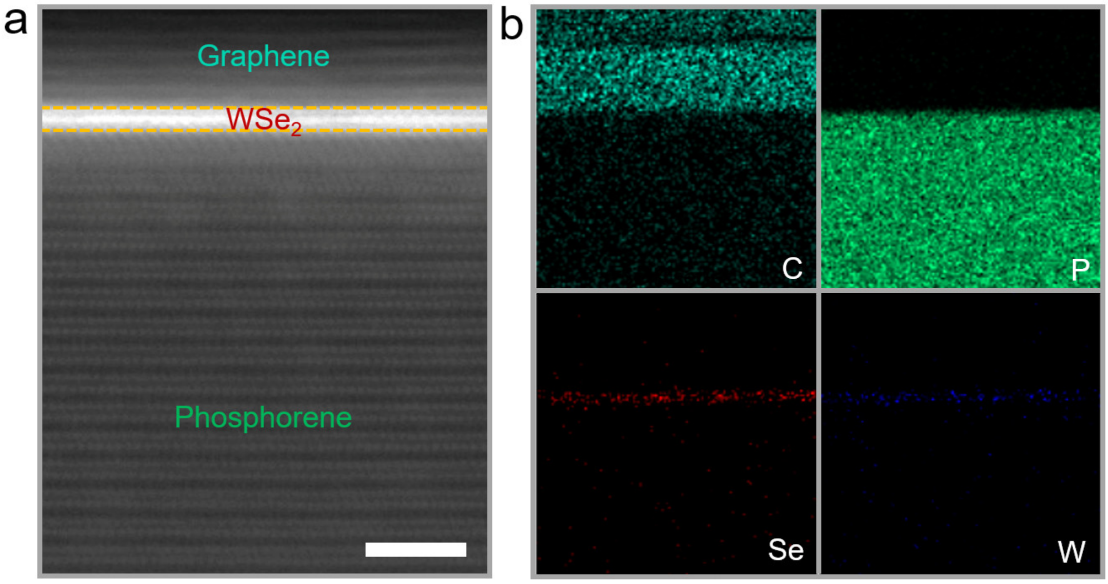

Supplementary Figure 2. **Cross-sectional characterizations of WSe_2/phosphorene interface. a** Cross-sectional scanning transmission electron microscopy (STEM) image of WSe_2/phosphorene interface. The scale bar is 2 nm. **b** Elemental mapping of WSe_2/phosphorene interface.

Supplementary Note 3

Based on the ISTD model, when $2 \le n \le N\text{-}1$, $\tau(n)$ satisfies the following equation when temperature changes from T_0 to T_1:

$$c_p\left(\Delta\tau(n+1)-\Delta\tau(n)\right)=\gamma_p\left(\tau(n)-\tau_p\right) \qquad 2\le n\le N\text{-}1 \tag{1}$$

Here, c_p is the interlayer coupling coefficient between phosphorene and phosphorene layers. γ_p and τ_p are Young's modulus and thermal-induced intrinsic deformation of phosphorene, respectively. τ_p is a constant depending on the TEC of phosphorene.

When n = 1, the STD of the bottom phosphorene is negligible from T_0 to T_1 due to the strong clamping effect and small TEC of SiO_2 substrates, which gives:

$$\tau(1)=0 \tag{2}$$

When n = N, the mechanical behaviors of the top phosphorene layers are totally different in phosphorene/SiO_2 and WSe_2/phosphorene/SiO_2 systems. For phosphorene/SiO_2, the N-th phosphorene merely interacts with the (N-1)-th phosphorene. Therefore, STD of the top layer, τ (N), satisfies the following equation at T_1:

$$-c_p\Delta\tau(N)=\gamma_p\left(\tau(N)-\tau_p\right) \tag{3}$$

Where $\Delta\tau(N)$ is the ISTD between the N-th and the (N-1)-th phosphorene. Then, τ (n) in phosphorene/SiO_2 can be obtained by solving Supplementary Equation (1), (2) and (3).

For WSe_2/phosphorene/SiO_2, considering the interlayer interactions between the top phosphorene and WSe_2, the STD of WSe_2, τ_h (N), satisfies Supplementary Equation (4) at T_1:

$$c_h\left(\tau(N)-\tau_h(N)\right)=\gamma_{WSe_2}(\tau_h(N)-\tau_{WSe_2}) \tag{4}$$

Here, c_h is the interlayer coupling coefficient between WSe_2 and phosphorene layers. γ_{WSe_2} is the Young's modulus of monolayer WSe_2. τ_{WSe_2} is the thermally-induced intrinsic in-plane deformation of monolayer WSe_2 from T_0 to T_1. Since the top phosphorene layer interacts with WSe_2 as well as the (N-1)-th phosphorene layer, $\tau(N)$ satisfies Supplementary Equation (5) at T_1:

$$c_h\left(\tau_h(N)-\tau(N)\right)-c_p\Delta\tau(N)=\gamma_p(\tau(N)-\tau_p) \tag{5}$$

Then c_p, τ_p, and $\tau_h(N)$ in WSe_2/phosphorene/SiO_2 can be obtained by solving Supplementary Equation (1), (2), (4) and (5).

Supplementary Note 4

A local band average approach under Debye approximation has declared that the temperature-dependent thermal expansion coefficient (TEC) is proportional to the specific heat (C_v) and can be expressed as[3]:

$$\alpha(T)=A(T/\theta_D)^3\int_0^{\theta_D/T}\frac{x^4e^x}{(e^x-1)^2}dx \tag{6}$$

Where A is a constant. Then the thermally-induced intrinsic strain of the monolayer WSe_2 can be obtained by integrating TEC:

$$\varepsilon(T)=\int\alpha(T)dT \tag{7}$$

In this work, 300 K is taken as the initial temperature T_0 and 10 K as the final temperature T_1. According to previous reports, the Debye temperature of atomically thin WSe_2 is around 170 K[4] and the TEC at 300 K[5-8] is around 7×10^{-6} K^{-1}. Utilizing

Supplementary Equation (6) and (7), the thermally-induced intrinsic deformation of WSe_2, τ_{WSe_2}, can be deduced as -0.17% from 300 to 10 K.

The value of c_h can be directly calculated utilizing the experiment data of WSe_2/1L phosphorene heterostructure and Supplementary Equation (4). Here, γ_{WSe_2} is 120 GPa [5, 9-12] and τ_{WSe_2} is -0.17%. When N = 1, τ (1) of phosphorene is 0 according to Supplementary Equation (2). The STD of WSe_2, τ_h (1), is measured as -0.054% at 10 K. Based on the above, the interlayer coupling coefficient c_h between WSe_2 and phosphorene is extracted as 2.72 $\times 10^{11}$ Pa.

Supplementary Note 5

We have performed experiments to investigate the temperature dependence of the strain gauge factor (η) for monolayer WSe_2. In order to measure the strain gauge factor, we exfoliate monolayer WSe_2 directly onto the polyimide (PI) membrane covered by a 50 nm-thick gold film (Supplementary Fig. 3a). We choose PI instead of PET membrane as the flexible substrate because PI can function well at low temperatures. Here, the 50 nm-thick gold film is sputtered onto PI membrane to eliminate the strong PL background from PI. Then, the WSe_2/Au/PI sample is loaded on a home-made strain setup with a two-point bending geometry, as shown in Supplementary Fig. 3b. Through pushing the side screw, the slider will move forward and PI membrane will be bend. A uniaxial tensile strain (ε) is therefore transferred to the monolayer WSe_2, which depends on the thickness (t = 200 μm) of substrate and the radius of curvature (R), $\varepsilon = t/2R$.

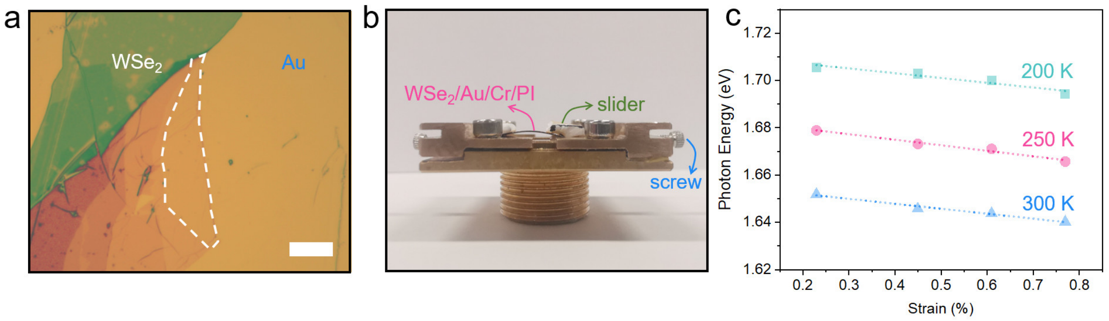

Supplementary Figure 3. Strain gauge factor of WSe_2. a Optical image of a monolayer WSe_2 on Au/PI membrane. The scale bar is 10 μm. **b** Optical image of the experimental setup. **c** Experimental measured (scatters) and theoretically fitted (dotted line) phonon energy of WSe_2 as a function of uniaxial tensile strain at 200, 250 and 300 K.

The strain setup is loaded in a He-flow closed-cycle cryostat with a high vacuum of ~2 × 10^{-6} Torr to conduct PL measurements. Noting that a severe PL quenching occurs when the monolayer WSe_2 is transferred onto Au layer. Besides, the PL intensity of monolayer WSe_2 decreases rapidly with temperature decreasing. As a result, the PL signal of neutral exciton can only be distinguished above 200 K in our experiments. Hence, we can only provide the data above 200 K and we sincerely apologize for this. We plot the photon energy of WSe_2 as a function of tensile strain (0.23%, 0.45%, 0.61%, 0.77%) at 200, 250, and 300 K, respectively (scatters in Supplementary Fig. 3c). The fitting results show clear linear dependence between photon energy and strain (dotted line in Supplementary Fig. 3c). The slope is extracted as -0.020, -0.023 and -0.021, based on which the biaxial strain gauge factor can be estimated as -40, -46 and -42 meV/% at 200, 250 and 300 K, respectively. Hence, this experiment demonstrates that

the strain gauge factor of WSe_2 shows weak temperature dependence, at least, in the range from 200 to 300 K.

	Ref.[13]	Ref.[14]	Ref.[15]	Ref.[16]	This work
Uniaxial (meV/%)	-54 (experiment)	–	–	-54 (experiment)	-21 (experiment)
Biaxial (meV/%)	-108 (estimated)	-63 (experiment) -134 (theory)	-105 (estimated)	-108 (estimated)	-42 (estimated)

Supplementary Table 1. Strain gauge factor of WSe_2 in previous reports and in this work.

Here, we list the strain gauge factor of WSe_2 obtained from our experiment and previous reports at room temperature in Supplementary Table 1. It is clear that our experimental results are smaller than previously reported values, which could be attributed to the inefficient strain transfer at the WSe_2/Au interface. The real strain gauge factor is always underestimated in experiments. Therefore, in the manuscript, the biaxial strain gauge factor is adopted as -100 meV/%, which stands between the experimental and theoretical values. To further study the influence of strain gauge factor to the fitting results, the strain gauge factor is set to be -80, -100 and -120 meV/%. As shown in Supplementary Table 2, both the interlayer coupling coefficients at WSe_2/phosphorene (c_h) and phosphorene/phosphorene (c_p) interfaces increase with $|\eta|$. Besides, c_h shows stronger η dependence than c_p. On the contrary, the intrinsic thermal deformation $|\tau_p|$ and TEC of phosphorene decrease with $|\eta|$.

η (meV/%)	c_h (Pa)	c_p (Pa)	τ_p	TEC at 300 K (K^{-1})
-80	1.94×10^{11}	3.22×10^{11}	-0.1 %	6.38×10^{-5}
-100	2.72×10^{11}	3.41×10^{11}	-0.711 %	4.52×10^{-5}
-120	3.50×10^{11}	3.63×10^{11}	-0.54 %	3.45×10^{-5}

Supplementary Table 2. Fitting results when the biaxial strain gauge factor of WSe_2 is set to be -80, -100 and -120 meV/%, respectively.

Supplementary Note 6

In the manuscript, the theoretical model is established through taking account into the intrinsic thermal deformation of phosphorene (τ_p), interlayer coupling coefficient at WSe_2/phosphorene (c_h) and phosphorene/phosphorene (c_p) interfaces. Here, c_h is directly calculated as 2.72×10^{11} Pa, while $c_p = 3.41\times10^{11}$ Pa and τ_p = -0.71 % are extracted through model fitting (using the least-square method). According to the standard deviation (0.0006) of fitted τ_h, upper and lower bounds of c_p and τ_p can be determined and are listed in Supplementary Table 3. Supplementary Fig. 4 further shows the fitting results at upper and lower bonds of c_p and τ_p.

	Fitted value	Upper bound	Lower bound
c_p	3.41×10^{11} Pa	4.80×10^{11} Pa	2.39×10^{11} Pa
τ_p	-0.71%	-0.65 %	-0.77 %

Supplementary Table 3. Parameters of c_p and τ_p.

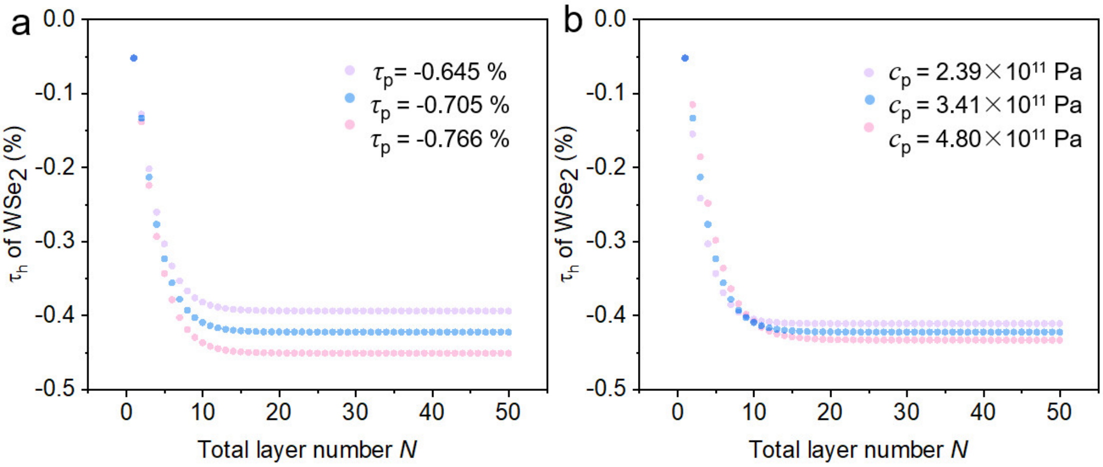

Supplementary Figure 4. **Error analysis of model fitting. a** Theoretically fitted shear thermal deformation (τ_h) of WSe_2 as a function of N at upper and lower bonds of τ_p. **b** Theoretically fitted τ_h of WSe_2 as a function of N at upper and lower bonds of c_p.

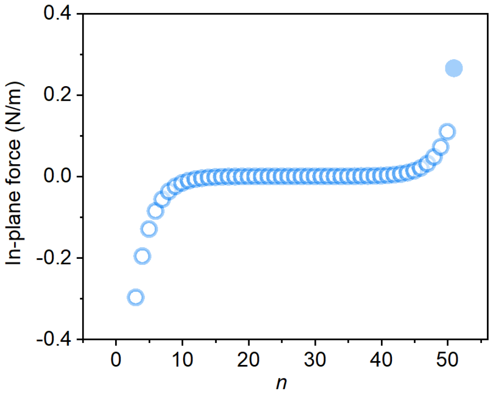

Supplementary Figure 5. **In-plane force in WSe_2/50-layer-phosphorene/SiO_2 system.** Layer-dependent in-plane force in phosphorene (hollow circles) and WSe_2 (solid circle) layers extracted from our model.

Supplementary References

1. Robertson, J. High dielectric constant oxides. *Eur. Phys. J.: Appl. Phys.* **28**, 265-291 (2004).

2. Qiu, D. Y., da Jornada, F. H. & Louie, S. G. Environmental screening effects in 2D materials: renormalization of the bandgap, electronic structure, and optical spectra of few-layer black phosphorus. *Nano Lett.* **17**, 4706-4712 (2017).

3. Gu, M., Zhou, Y. & Sun, C. Q. Local bond average for the thermally induced lattice expansion. *J. Phys. Chem. B* **112**, 7992-7995 (2008).

4. Arora, A. et al. Excitonic resonances in thin films of WSe_2: from monolayer to bulk material. *Nanoscale* **7**, 10421-10429 (2015).

5. Çakır, D., Peeters, F. M. & Sevik, C. Mechanical and thermal properties of h-MX_2 (M = Cr, Mo, W; X = O, S, Se, Te) monolayers: A comparative study. *Appl. Phys. Lett.* **104**, 203110 (2014).

6. Wang, Z.-Y. et al. Effects of in-plane stiffness and charge transfer on thermal expansion of monolayer transition metal dichalcogenide. *Chin. Phys. B* **24**, 026501 (2015).

7. Morell, N. et al. High quality factor mechanical resonators based on WSe_2 monolayers. *Nano Lett.* **16**, 5102-5108 (2016).

8. Li, Z. et al. Temperature-dependent Raman spectroscopy studies of 1–5-layer WSe_2. *Nano Res.* **13**, 591-595 (2020).

9. Feng, L.-p., Li, N., Yang, M.-h. & Liu, Z.-t. Effect of pressure on elastic, mechanical and electronic properties of WSe_2: A first-principles study. *Mater. Res. Bull.* **50**, 503-508 (2014).

10. Zeng, F., Zhang, W.-B. & Tang, B.-Y. Electronic structures and elastic properties of monolayer and bilayer transition metal dichalcogenides MX_2 (M = Mo, W; X = O, S, Se, Te): A comparative first-principles study. *Chin. Phys. B* **24**, 097103 (2015).

11. Kang, J., Tongay, S., Zhou, J., Li, J. & Wu, J. Band offsets and heterostructures of two-dimensional semiconductors. *Appl. Phys. Lett.* **102**, 012111 (2013).

12. Ding, W., Han, D., Zhang, J. & Wang, X. Mechanical responses of WSe_2 monolayers: a molecular dynamics study. *Mater. Res. Express* **6**, 085071 (2019).

13. Schmidt, R. et al. Reversible uniaxial strain tuning in atomically thin WSe_2. *2D Mater.* **3**, 021011 (2016).

14. Frisenda, R. et al. Biaxial strain tuning of the optical properties of single-layer transition metal dichalcogenides. *npj 2D Mater. Appl.* **1**, 10 (2017).

15. Ahn, G. H. et al. Strain-engineered growth of two-dimensional materials. *Nat. Commun.* **8**, 608 (2017).

16. Cho, C. et al. Highly strain-tunable interlayer excitons in MoS_2/WSe_2 heterobilayers. *Nano Lett.* **21**, 3956-3964 (2021).
